# Killing a Rhinoceros

**Published:** 1882-05

**Authors:** 


### KILLING A RHINOCEROS.

During a trip to Boerland, South Africa, an English gentleman and his friend had the glory of shooting a rhinoceros.

The encounter with the huge, unwieldy beast occurred at night, by a water-hole, while they were waiting for game. Each of the Englishmen was seated with a Kaffir servant in the fork of a tree looking out for buffalo. There were plenty of bush-buck and other antelope drinking and gamboling at the edge of the water-hole, but the sportsmen were determined to shoot at nothing less than a buffalo.

At last, the advance of two magnificent koodoos, whose beautiful spiral horns towered above them as they walked into the pool, caused the sportsmen to forget their resolution. Both fired. One koodoo fell; the other made off desperately wounded.

One of the Englishmen was about descending the tree. A shout from his friend, and a heavy footfall, accompanied with a puff, puff, like that of a steam engine, warned him to remain. Out from the dense bush came a black rhinoceros, mad with wickedness. Seeing the hunters, he butted again and again the trees in which they were seated. His furious charges almost shook them off. Spiteful at not being able to get at them, the animal plowed up the ground with his horn.

Bullet after bullet was fired into him, but, apparently, he was as indifferent as if he had been hit with peas. At length an expansive ball dropped on his snout, just behind his horn, and brought him to his knees. Getting up and stamping the ground, he made for the bush. At daylight the hunters followed his trail, which at first was marked with blood. About a mile, on the flattened grass showed where the brute had rested. The trail led across an open sward of grass into a thicket. Just as the hunters were cautiously entering it, a bird flew up, uttering a sharp "Tcha, tcha!" Out came the beast, staggering as if drunk. Three shots brought him to his knees. But up he rose, faced round, as if to charge, changed his mind, and ran into the bush. The hunters, following, found him lying on his side, and quite dead. The wonderful vitality of the animal is shown in the fact that he had lived several hours with thirteen bullets in his body, and after trotting three or four miles, and bleeding profusely, had retained his life until three more shots killed him.

